# Nested Variational Chain and Its Application in Massive MIMO Detection for High-Order Constellations

**DOI:** 10.3390/e25121621

**Published:** 2023-12-05

**Authors:** Qiwei Wang

**Affiliations:** School of Telecommunications Engineering, Xidian University, Xi’an 710071, China; qwwang@xidian.edu.cn

**Keywords:** massive multiple input multiple output (MIMO), nested variational chain, Gaussian tree approximation (GTA), expectation consistency (EC)

## Abstract

Multiple input multiple output (MIMO) technology necessitates detection methods with high performance and low complexity; however, the detection problem becomes severe when high-order constellations are employed. Variational approximation-based algorithms prove to deal with this problem efficiently, especially for high-order MIMO systems. Two typical algorithms named Gaussian tree approximation (GTA) and expectation consistency (EC) attempt to approximate the true likelihood function under discrete finite-set constraints with a new distribution by minimizing the Kullback–Leibler (KL) divergence. As the KL divergence is not a true distance measure, ’exclusive’ and ’inclusive’ KL divergences are utilized by GTA and EC, respctively, demonstrating different performances. In this paper, we further combine the two asymmetric KL divergences in a nested way by proposing a generic algorithm framework named nested variational chain. Acting as an initial application, a MIMO detection algorithm named Gaussian tree approximation expectation consistency (GTA-EC) can thus be presented along with its alternative version for better understanding. With less computational burden compared to its counterparts, GTA-EC is able to provide better detection performance and diversity gain, especially for large-scale high-order MIMO systems.

## 1. Introduction

Multiple input multiple output (MIMO) technology has attracted broad attention over the last decade and has been widely applied into practical communication systems. The benefit of MIMO technology lies in the improvement of spectral efficiency and link reliability due to the multiplexing and diversity gain that grows with the number of elements, and a MIMO system is referred to as a *massive* MIMO system when the scale of array elements grows large enough, which brings increasing difficulty to the signal detection due to huge computational burden, hindering the prevailing usage of massive MIMO systems [1,2].

Many research studies have been carried out for signal detection in massive MIMO systems [3,4,5,6,7,8,9,10,11,12,13,14,15,16,17]. It is well known that the maximum likelihood detection presents the best detection performance with the cost of exponentially growing computational burden [3]. Neglecting the finite-set constraint, the minimum mean square error (MMSE) approach can be applied by solving the least square fit, and a closest lattice point can then be found by treating symbols independently [4]. The MMSE approach normally exhibits a benchmark performance when comparing different detectors, and its performance can be vastly improved by MMSE-SIC when combining with the successive interference cancellation (SIC) technique [5]. However, as MMSE or MMSE-SIC cannot provide satisfied performance, several alternatives have been proposed instead, which can be divided into two major categories, i.e., sub-space searching-based and variational inference-based detectors.

The sub-space searching-based category originates from the idea of reducing the searching space of all possible lattice points with unacceptable complexity. Sphere decoding tries to replicate the maximum likelihood performance by diminishing the searching space, the dimension of which grows up with the number of antennas as well as the modulation order, making it prohibitive for the large-scale or high-order MIMO systems [6,7]. Another two local searching-based approaches were proposed by the name of likelihood ascending search and reactive tabu search [8,9,10], and the basic idea behind them is to search through a proximity sub-space around a given initial solution. They present good performance for a large number of antennas with low-order constellations but poor performance for high-order constellations. A layered tabu search algorithm was proposed in [11] by performing detection over layers, requiring a higher order of complexity for high-order constellations, and a Gibbs sampling-based detector was proposed in [12] by performing a serial of one-dimensional searches over iterations. It may provide good performance for low-order constellations with the cost of enormous processing time. Therefore, algorithms in the former category suffer from poor performance or prohibitive computational burden in large-scale high-order MIMO systems.

Proved to be suitable for the detection problem resulted by high-order constellations, the variational inference based category tries to approximate the true likelihood function into a new distribution that is much easier to handle. A Gaussian tree approximation (GTA) algorithm was proposed in [13,14] by transforming the fully connected factor graph into a tree graph, based on which belief propagation based message passing can be proceeded for inference. The GTA algorithm has comparable performance with MMSE-SIC at a similar complexity only to MMSE. The expectation propagation (EP) algorithm was proposed for MIMO detection in [15] by substituting true priors belonging to a discrete finite set with the introduced Gaussian priors being able to be updated over iterations. EP performs the best at a complexity several times that of MMSE, and its alternative named expectation consistency (EC) was then proposed to provide a more general perspective than EP [16]. Two low-complexity EP/EC-based algorithms were proposed for scenarios when the number of transmit antennas is less than that the number of receiver ones [17,18], and a double-EP based iterative detection and decoding was proposed by iteratively exploiting decoders in [19]. EP/EC-based algorithms can also be applied into channel estimation problems in massive MIMO systems [20,21].

In this paper, we would like to expand the variational inference paradigm by proposing a nested variational chain. The basic idea behind it is that ’exclusive’ and ’inclusive’ KL divergences employed by GTA and EC, respectively, are not exclusive and can be combined in a nested way so as to form an approximation chain, by which both GTA and EC are improved. The major contributions are listed as follows.

- Firstly, the basic idea of the nested variational chain is proposed, and an algorithm is then proposed to establish a general framework. By referring to ’general’, it means this framework is able to combine ’exclusive’ and ’inclusive’ KL divergences, or it degrades to either one as a special case.
- Secondly, providing several examples, we show that existing algorithms, such as MMSE, GTA, and EC, can be regarded as special cases of the variational chain.
- Finally, to provide an initial application of the variational chain into massive MIMO detection, a GTA-embedded Expecatation Consistency (GTA-EC) algorithm is proposed which proves to provide better detection performance, especially for high-order constellations. The complexity of GTA-EC is analyzed as well along with comparisons.

This paper is arranged as follows. Section 2 introduces the system model and MIMO detection problem, based on which the nested variational chain is provided in Section 3 along with a generic algorithm framework. Section 4 derives the GTA-EC algorithm with complexity analyses. Simulation results are demonstrated in Section 5 along with discussions, and the conclusion is presented in Section 6. Throughout this paper, matrices and vectors are denoted by symbols in boldface, and variables are denoted in italics. The notation $A^{⊤}$ or $a^{⊤}$ is used to represent the transpose of a vector or matrix, and I represents a unit matrix.

## 2. Preliminary

### 2.1. Signal Model

A multiuser MIMO system is considered, without loss of generality, in which $\tilde{N}$ transmitters, each equipped with one antenna, communicate with a base station that is equipped with $\tilde{M}$ antennas. Assume each transmitter transmits any symbol ${\tilde{x}}_{i}\in C$ that is selected from a Quadrature Amplitude Modulation (QAM) constellation set $\tilde{A}$, where C stands for the complex domain, and the cardinality of the constellation set is $|\tilde{A}|=A$. The transmitted symbols can be represented as a vector $\tilde{x}\in {\tilde{A}}^{\tilde{N}\times 1}$ with the average energy of each QAM symbol defined as ${\tilde{E}}_{s}$. After propagating through the wireless channels, the received signal $\tilde{y}\in C^{\tilde{M}\times 1}$ at the base station can be expressed as

(1) $$\begin{array}{c} \tilde{y}=\tilde{H}\;\tilde{x}+\tilde{n}, \end{array}$$

where $\tilde{n}\in C^{M\times 1}$ stands for additive Gaussian white noises (AWGN), each element having zero mean and $\sigma_{n}^{2}$ variance. $\tilde{H}≜\left[{\tilde{h}}_{1},\ldots ,{\tilde{h}}_{i},\ldots ,{\tilde{h}}_{N}\right]$ is defined as a matrix by stacking up channel coefficients with ${\tilde{h}}_{i}=\mathrm{diag}\left\{{\tilde{h}}_{i,1},\ldots ,{\tilde{h}}_{i,m},\ldots ,{\tilde{h}}_{i,M}\right\}$ being Rayleigh flat-fading channel coefficients of the $i^{}$th symbol. Perfect channel state information (CSI) is assumed such that $\tilde{H}$ is known at the base station.

The channel model above is usually re-expressed in the real domain by taking into consideration real and imaginary parts, respectively. By defining R(·) and I(·) as operations to take the real and imaginary part of a variable or matrix, one can define $y={\left[R{\left(\tilde{y}\right)}^{⊤}\;I{\left(\tilde{y}\right)}^{⊤}\right]}^{⊤}$, $x={\left[R{\left(\tilde{x}\right)}^{⊤}\;I{\left(\tilde{x}\right)}^{⊤}\right]}^{⊤}$, $n={\left[R{\left(\tilde{n}\right)}^{⊤}\;I{\left(\tilde{n}\right)}^{⊤}\right]}^{⊤}$, and that

$$\begin{array}{c} H=\left[\begin{array}{cc} R(\tilde{H}) & -I(\tilde{H})\\ I(\tilde{H}) & \;R(\tilde{H}) \end{array}\right], \end{array}$$

where $y,n\in R^{M\times 1}$, $x\in R^{N\times 1}$, $H\in R^{M\times N}$, $M=2\tilde{M}$, $N=2\tilde{N}$, and R stands for the real domain. The equivalent model in the real domain is then given as

(2) $$\begin{array}{c} y=H\;x+n, \end{array}$$

where the variance of each element of n equals $\sigma_{n}^{2}={\tilde{\sigma }}_{n}^{2}/2$, and x belongs to the pulse amplitude modulation (PAM) constellation set A containing real and imaginary parts of the A-QAM alphabets with its cardinality being $\left|A\right|=\sqrt{A}$. The average energy of a PAM symbol is $E_{s}={\tilde{E}}_{s}/2$, and the signal-to-noise (SNR) of the MIMO system is then defined as

$$\begin{array}{c} \mathrm{SNR}=10\cdot \log_{10}\left(\frac{N{\tilde{E}}_{s}}{{\tilde{\sigma }}_{n}^{2}}\right)=10\cdot \log_{10}\left(\frac{NE_{s}}{\sigma_{n}^{2}}\right) \end{array}$$

### 2.2. MIMO Detection

As the received signal in a MIMO system is a superposition of transmitted symbols weighted by channel coefficients, the purpose of MIMO detection is to estimate successfully all transmitted symbols impaired by channel fading and noises. As is well known, the maximum *a posteriori* (MAP) detector could achieve the best detection performance by maximizing the a posteriori probability as follows

$$\begin{array}{c} \hat{x}=\underset{x\in A}{\arg\max}P\left(x|y,H\right), \end{array}$$

where the aposteriori distribution given the received signal y and CSI H is expressed as

(3) $$\begin{array}{cc} P\left(x|y,H\right) & \propto N\left(y:\mathrm{Hx},\sigma_{n}^{2}I\right)P\left(x\right), \end{array}$$

$N\left(y:\mathrm{Hx},\sigma_{n}^{2}I\right)$ is defined as Gaussian distribution with a mean vector of Hx and a covariance matrix of $\sigma_{n}^{2}I$, and $P\left(x\right)$ is defined as the a priori probability of symbols. When $P\left(x\right)=\prod_{i=1}^{N}\frac{1}{\sqrt{A}}I_{x_{i}\in A}$ is uniformly distributed with $I_{x_{i}\in A}$ being an indication function that takes value one if $x_{i}\in A$ and zero otherwise, the MAP detection degrades into the maximum likelihood detection, i.e.,

$$\begin{array}{c} \hat{x}=\underset{x}{\arg\max}N\left(y:\mathrm{Hx},\sigma_{n}^{2}I\right)\prod_{i=1}^{N}I_{x_{i}\in A} \end{array}$$

The complexity of maximum likelihood detection grows up exponentially with the number of symbols *N*, making it prohibitive for middle- or large-scale MIMO systems with especially high-order constellations.

## 3. Nested Variational Chain

In order to perform low-complexity MIMO detection, one popular approach is to approximate the true posterior with another distribution that is much simpler to perform inference on, and the KL divergence is commonly used to obtain the desired distribution. Defining $Q\left(x\right)$ as the distribution utilized to approximate the true posterior, the minimization of the ’exclusive’ and ’inclusive’ KL divergences can be expressed as

(4) $$\begin{array}{cc} Q\left(x\right) & =\underset{Q^{\prime }\left(x\right)}{\arg\min}\mathrm{KL}\left(Q^{\prime }\left(x\right)\left|\right|P\left(x|y,H\right)\right)\\ \; & =\underset{Q^{\prime }\left(x\right)}{\arg\min}\int_{x}Q^{\prime }\left(x\right)\log\frac{Q^{\prime }\left(x\right)}{P\left(x|y,H\right)}, \end{array}$$

and

(5) $$\begin{array}{cc} Q\left(x\right) & =\underset{Q^{\prime }\left(x\right)}{\arg\min}\mathrm{KL}\left(P\left(x|y,H\right)\left|\right|Q^{\prime }\left(x\right)\right)\\ \; & =\underset{Q^{\prime }\left(x\right)}{\arg\min}\int_{x}P\left(x|y,H\right)\log\frac{P\left(x|y,H\right)}{Q^{\prime }\left(x\right)}. \end{array}$$

For instance, the GTA algorithm takes the former way, while the EC algorithm takes the latter one. However, with only one approximation, GTA is unable to update its approximated tree structure, while EC only treats symbols in an independent way rather than exploiting correlation among symbles. In this case, we then would like to demonstrate that the two KL divergences could be combined together, and a nested variational chain is then proposed in what follows.

Suppose there is a desired variational distribution $G\left(x\right)$ that can be obtained with ’exclusive’ KL divergengce:

(6) $$\begin{array}{c} G\left(x\right)=\underset{G^{\prime }\left(x\right)}{\arg\min}\mathrm{KL}\left(Q\left(x\right)\left|\right|G^{\prime }\left(x\right)\right), \end{array}$$

which is embedded in an optimization for $Q\left(x\right)$ with $Q\left(x\right)$ obtained in the first place as

(7) $$\begin{array}{c} Q\left(x\right)=\underset{Q^{\prime }\left(x\right)}{\arg\min}\mathrm{KL}\left(P\left(x|y,H\right)\left|\right|Q^{\prime }\left(x\right)\right). \end{array}$$

The processing above actually forms a variational chain with a nested structure given as

(8) $$\begin{array}{c} P\left(x|y,H\right)\Rightarrow Q\left(x\right)\Rightarrow G\left(x\right), \end{array}$$

indicating $Q\left(x\right)$ should be obtained according to the minimization of $\mathrm{KL}\left(P\left(x|y,H\right)\left|\right|Q^{\prime }\left(x\right)\right)$ with respect to $Q^{\prime }\left(x\right)$, and that the desired $G\left(x\right)$ could then be obtained by minimizing $\mathrm{KL}\left(Q\left(x\right)\left|\right|G^{\prime }\left(x\right)\right)$ with respect to $G^{\prime }\left(x\right)$. Following the roadmap, we may derive an algorithm for the nested variational chain combining the two asymetric KL divergences.

### 3.1. A Generic Framework for Nested Variational Chain

To begin with, a general statistical model should first be defined as follows [12],

(9) $$\begin{array}{c} P\left(x\right)\propto F\left(x\right)\prod_{i}t_{i}\left(x\right), \end{array}$$

where $F\left(x\right)$ is a function belonging to the exponential family, and $t_{i}\left(x\right)$ for i=1,…,I are non-negative factors. Normally, it is intractable or prohibitive complex to perform inference over $P\left(x\right)$ such that the variational inference-based approaches provide another distribution $Q\left(x\right)$ that is tractable or easy to handle. The nested variational chain consists of four steps: factor substitution, inner approximation, symbol detection and factor updating.

As for factor substitution, the optimization of the KL divergence should first be achieved, i.e., $Q\left(x\right)={\arg\min}_{Q^{\prime }\left(x\right)}\mathrm{KL}\left(P\left(x\right)\left|\right|Q^{\prime }\left(x\right)\right)$, for which the EC framework can be employed. The EC algorithm assumes a distribution that belongs to the exponential family:

(10) $$\begin{array}{c} Q\left(x\right)\propto F\left(x\right)\prod_{i}{\tilde{t}}_{i}\left(x\right), \end{array}$$

where ${\tilde{t}}_{i}\left(x\right)$ instead of $t_{i}\left(x\right)$ for i=1,…,I are modified factors, belonging to the exponential family as well.

It should be noticed that the EC framework replaces each non-negative factor $t_{i}\left(x\right)$ by another ${\tilde{t}}_{i}\left(x\right)$ in the exponential family. However, the distribution $F\left(x\right)$ remains constant during this optimization process, which may be further exploited. Based on this idea, another variational distribution could be embedded inside as an inner approximation so as to achieve a final distribution $G\left(x\right)$, and the optimization in (6) could be performed:

(11) $$\begin{array}{cc} G\left(x\right) & =\underset{G^{\prime }\left(x\right)}{\arg\min}\mathrm{KL}\left(G^{\prime }\left(x\right)\left|\right|Q\left(x\right)\right)\\ \; & =\underset{G^{\prime }\left(x\right)}{\arg\min}\mathrm{KL}\left(G^{\prime }\left(x\right)\left|\right|F\left(x\right)\prod_{i}{\tilde{t}}_{i}\left(x\right)\right). \end{array}$$

The approximation is normally expressed as $G\left(x\right)=\prod_{j}G_{j}\left(x\right)$. When $G_{j}\left(x\right)$ for j=1,…,J are defined as disjoint groups, it is mean-field approximation, and structured approximation can be employed when $G_{j}\left(x\right)$ for j=1,…,J are overlapped with each other.

Toward symbol detection, a cavity distribution for each factor can then be acquired as

(12) $$\begin{array}{c} G^{\setminus i}\left(x\right)=\frac{G\left(x\right)}{{\tilde{t}}_{i}\left(x\right)} \end{array}$$

and the final distribution can be represented as

(13) $$\begin{array}{c} {\tilde{G}}_{i}\left(x\right)\propto \prod_{i=1}^{N}\underset{p_{i}\left(x\right)}{\underset{︸}{G^{\setminus i}\left(x\right)t_{i}\left(x\right)}}, \end{array}$$

with $p_{i}\left(x\right)≜G^{\setminus i}\left(x\right)t_{i}\left(x\right)$ defined as a new distribution by attaching the true factor.

The moments of $p_{i}\left(x\right)$ are then obtained by exploiting the true distribution $t_{i}\left(x\right)$ as $E_{p_{i}\left(x\right)}\left[\phi \left(x\right)\right]$, where $\phi \left(x\right)$ stands for the sufficient statistics of the exponential family. A new factor ${\tilde{t}}_{i}^{\;new}\left(x\right)$ is updated as well by satisfying the moment-matching condition $E_{p_{i}\left(x\right)}\left[\phi \left(x\right)\right]=E_{q_{i}\left(x\right)}\left[\phi \left(x\right)\right]$ with

(14) $$\begin{array}{c} q_{i}\left(x\right)\propto G^{\setminus i}\left(x\right){\tilde{t}}_{i}^{\;new}\left(x\right), \end{array}$$

such that the distribution $Q\left(x\right)$ is able to be updated iteratively.

An algorithm is provided in Algorithm 1, which is used to approximate a statistical model $P\left(x\right)\propto F\left(x\right)\prod_{i}t_{i}\left(x\right)$. Associating with the four steps described above, the algorithm first substites all factors in Step 1 with much easier accessible ones by using the ’inclusive’ KL divergence, as seen in the EC algorithm. After that, the algorithm further approximates $Q\left(x\right)$ with a new distribution in Step 2 such that better detection performance is expected. With detection proceeded on the new distribution, moment matching can be achieved in Step 3 so as to update substituted factors in Step 4. Note that any of the steps, such as factor substitution, inner approximation, or factor updating, may be skipped for a certain purpose so as to form a special case. In the next subsection, we would like to demonstrate that the MMSE, GTA and EC algorithms could be deemed as special cases.
**Algorithm 1** An algorithm for nested variational chain**Require:** A statistical model $P\left(x\right)\propto F\left(x\right)\prod_{i}t_{i}\left(x\right)$.**Ensure:****repeat****(1) Step 1: Factor Substitution**.Substitute each non-negative factor $t_{i}\left(x\right)$ with ${\tilde{t}}_{i}\left(x\right)$ for i=1,…,I such that $Q\left(x\right)\propto F\left(x\right)\prod_{i}{\tilde{t}}_{i}\left(x\right)$.**(2) Step 2: Inner Approximation**.Obtain a new distribution to approximate $Q\left(x\right)$ as
$$\begin{array}{c} G\left(x\right)=\underset{G^{\prime }\left(x\right)}{\arg\min}\mathrm{KL}\left(G^{\prime }\left(x\right)\left|\right|Q\left(x\right)\right) \end{array}$$**(3) Step 3: Symbol Detection**.**for** $i\in \left[1,\ldots ,I\right]$ **do**Obtain a cavity distribution as $G^{\setminus i}\left(x\right)=G\left(x\right)\;{\tilde{t}}_{i}\left(x\right)$, and then achieve moment matching between $p_{i}\left(x\right)\propto G^{\setminus i}\left(x\right)t_{i}\left(x\right)$ and $q_{i}\left(x\right)\propto G^{\setminus i}\left(x\right){\tilde{t}}_{i}^{\;new}\left(x\right)$.**end for****(4) Step 4: Factor Updating**.Substitute ${\tilde{t}}_{i}\left(x\right)$ with ${\tilde{t}}_{i}^{\;new}\left(x\right)$ into $Q\left(x\right)\propto F\left(x\right)\prod_{i}{\tilde{t}}_{i}\left(x\right)$ and repeat this procedure if necessary.**until** Convergence is achieved.**Output:** Detection results on the approximated distribution.

### 3.2. MMSE, GTA and EC MIMO Detectors as Special Cases

In a MIMO system, the distribution $F\left(x\right)$ can be expressed as the likelihood function, i.e., $F\left(x\right)\propto N\left(y:\mathrm{Hx},\sigma_{n}^{2}I\right)$, and each non-negative factor $t_{i}\left(x\right)$ for i=1,…,I could be regarded as the apriori probability with respect to symbols. When a factor $t_{i}\left(x\right)$ corresponds only to one symbol $x_{i}$, it reduces to $t_{i}\left(x_{i}\right)=P\left(x_{i}\right)=\frac{1}{\sqrt{A}}I_{x_{i}\in A}$. Hence, as there are *N* symbols in a MIMO system, there would be *N* factors or priors as well, and the expression for the substituted factor ${\tilde{t}}_{i}\left(x_{i}\right)$ for i=1,…,N depends on any specific algorithm.

(1) Minimum Mean Square Error  

The MMSE approach could be obtained by assuming that each non-negative factor $t_{i}\left(x_{i}\right)$ for i=1,…,N can be replaced by a Gaussian distributed factor ${\tilde{t}}_{i}\left(x_{i}\right)=N\left(x_{i}:0,E_{s}\right)$ of zero-mean and a variance of $E_{s}$, and the modified distribution with factor substitution for MMSE is then given as

(15) $$\begin{array}{c} Q_{MMSE}\left(x\right)\propto N\left(y:\mathrm{Hx},\sigma_{n}^{2}I\right)\prod_{i=1}^{N}N\left(x_{i}:0,E_{s}\right), \end{array}$$

whose second-order and first-order moments are derived as

$$\left\{\begin{array}{cc} \; & \Sigma_{MMSE}={\left(H^{⊤}H+\frac{\sigma_{n}^{2}}{E_{s}}I\right)}^{-1}\\ \; & \mu_{MMSE}=\Sigma_{MMSE}H^{⊤}y \end{array}\right.$$

Not mentioned though before, there is actually a simple inner approximation for MMSE to approximate the distribution $Q_{MMSE}\left(x\right)$. With a fully factorized distribution $G_{MMSE}\left(x\right)=\prod_{i}^{N}G_{MMSE}\left(x_{i}\right)$, each factorized one can be obtained as

(16) $$\begin{array}{cc} G_{MMSE}\left(x_{i}\right) & \propto \exp\left({\langle \ln Q_{MMSE}\left(x\right)\rangle }_{∼G_{MMSE}\left(x_{i}\right)}\right)\\ \; & \propto N\left(x_{i}:\mu_{i,MMSE},\Sigma_{i,MMSE}\right), \end{array}$$

which is known as the mean-field approximation. The expression ${\langle \cdot \rangle }_{∼G_{MMSE}\left(x_{i}\right)}$ refers to expectation with respect to all factors $G_{MMSE}\left(x_{j}\right)$ for j=1,…,N except for $G_{MMSE}\left(x_{i}\right)$. This process is equivalent to marginalization of $Q_{\mathrm{MMSE}}\left(x\right)$ with $\mu_{i,MMSE}$ and $\Sigma_{i,MMSE}$ being the $i^{th}$ element of $\mu_{\mathrm{MMSE}}$ and of the diagonal of $\Sigma_{MMSE}$.

The MMSE approach skips factor updating, but instead it may output directly the hard detection results. The final distribution of MMSE is expressed as

(17) $$\begin{array}{c} {\tilde{G}}_{MMSE}\left(x\right)\propto \prod_{i=1}^{N}\underset{p_{MMSE,i}\left(x_{i}\right)}{\underset{︸}{G_{MMSE}\left(x_{i}\right)t_{i}\left(x_{i}\right)}}, \end{array}$$

where $p_{MMSE,i}\left(x_{i}\right)≜G_{MMSE}\left(x_{i}\right)t_{i}\left(x_{i}\right)$ is defined as a new distribution by attaching true priors, based on which symbol detection can be proceeded for each symbol independently.

(2) Expectation Consistency  

The EC algorithm defines a substitution factor for each symbol as well. It replaces the prior $t_{i}\left(x_{i}\right)=\frac{1}{\sqrt{A}}I_{x_{i}\in A}$ to ${\tilde{t}}_{i}\left(x_{i}\right)\propto e^{\gamma_{i}x_{i}-\frac{1}{2}\Lambda_{i}x_{i}^{2}}$ so the posterior can be expressed as

(18) $$\begin{array}{cc} Q_{EC}\left(x\right) & \propto N\left(y:\mathrm{Hx},\sigma_{n}^{2}I\right)\prod_{i=1}^{N}e^{\gamma_{i}x_{i}-\frac{1}{2}\Lambda_{i}x_{i}^{2}} \end{array}$$

Note that ${\tilde{t}}_{i}\left(x_{i}\right)\propto e^{\gamma_{i}x_{i}-\frac{1}{2}\Lambda_{i}x_{i}^{2}}$ is Gaussian distributed. In this regard, it can be noticed that EC relates essentially to MMSE with the difference that it is able to update priors. The second-order and first-order moments of $Q_{\mathrm{EC}}\left(x\right)$ are derived as

$$\left\{\begin{array}{cc} \; & \Sigma_{EC}={\left(\sigma_{n}^{-2}H^{⊤}H+\Lambda \right)}^{-1}\\ \; & \mu_{EC}=\Sigma_{EC}\left(H^{⊤}y+\gamma \right) \end{array}\right.$$

where Λ is a diagonal matrix containing $\Lambda_{i}$, and γ is a vector containing $\gamma_{i}$ for i=1,…,N.

The EC algorithm employs mean-field approximation for inner approximation as well, by which the fully factorized distribution is defined as $G_{EC}\left(x\right)=\prod_{i}^{N}G_{EC}\left(x_{i}\right)$, and each factorized distribution $G_{EC}\left(x_{i}\right)$ is Gaussian distributed such that:

(19) $$\begin{array}{cc} G_{EC}\left(x_{i}\right) & \propto \exp\left({\langle \ln Q_{EC}\left(x\right)\rangle }_{∼G_{EC}\left(x_{i}\right)}\right)\\ \; & \propto N\left(x_{i}:\mu_{i,EC},\Sigma_{i,EC}\right), \end{array}$$

with $\mu_{i,EC}$ and $\Sigma_{i,EC}$ being the $i^{th}$ element of $\mu_{EC}$ and of the diagonal of $\Sigma_{EC}$, respectively. By doing so, factor updating is then operated with a cavity distribution:

(20) $$\begin{array}{c} G_{EC}^{\setminus i}\left(x_{i}\right)=\frac{G_{EC}\left(x_{i}\right)}{{\tilde{t}}_{i}\left(x_{i}\right)}, \end{array}$$

and the final distribution for EC is represented as

(21) $$\begin{array}{c} {\tilde{G}}_{EC}\left(x\right)\propto \prod_{i=1}^{N}\underset{p_{EC,i}\left(x_{i}\right)}{\underset{︸}{G_{EC}^{\setminus i}\left(x_{i}\right)t_{i}\left(x_{i}\right)}}, \end{array}$$

where $p_{EC,i}\left(x_{i}\right)≜G_{EC}^{\setminus i}\left(x_{i}\right)t_{i}\left(x_{i}\right)$ is defined as a new distribution. Symbol detection can then be performed to achieve the moment-matching condition so the pairs $\left(\gamma_{i},\Lambda_{i}\right)$ for i=1,…,N are updated in parallel.

(3) Gaussian Tree Approximation  

The GTA algorithm was proposed based on the modified distribution of MMSE, and its distribution with substituted factors can be represented as

(22) $$\begin{array}{cc} Q_{GTA}\left(x\right) & =Q_{MMSE}\left(x\right)\propto F\left(x\right)\prod_{i=1}^{N}{\tilde{t}}_{i}\left(x_{i}\right)\\ \; & \propto N\left(y:\mathrm{Hx},\sigma_{n}^{2}I\right)\prod_{i=1}^{N}N\left(x_{i}:0,E_{s}\right). \end{array}$$

As for inner approximation, the GTA algorithm chooses to optimally approximate the distribution with a tree graph, which can be constructed based on $Q_{GTA}\left(x\right)$ as

(23) $$\begin{array}{cc} G_{GTA}\left(x\right) & =\underset{G^{\prime }\left(x\right)}{\arg\min}\mathrm{KL}\left(G^{\prime }\left(x\right)\left|\right|Q_{GTA}\left(x\right)\right)\\ \; & =\prod_{i}G_{GTA}\left(x_{i}|x_{pa(i)}\right), \end{array}$$

where $G_{GTA}\left(x_{i}|x_{pa(i)}\right)$ stands for the conditional probability of $x_{i}$ given its parent $x_{pa(i)}$, and $G_{GTA}\left(x_{i}|x_{pa(i)}\right)=G_{GTA}\left(x_{i}\right)$ in case that $x_{i}$ is the root of the tree.

This leads to a result that GTA skips factor updating as well, similar to MMSE, and the performance of the GTA algorithm is subject to the fixed initial distribution $Q_{GTA}\left(x\right)$ that is not able to be updated. In this case, by directly attaching the true priors $t_{i}\left(x_{i}\right)=\frac{1}{\sqrt{A}}I_{x_{i}\in A}$ for i=1,…,N, the final distribution of GTA is then represented as

(24) $$\begin{array}{cc} {\tilde{G}}_{GTA}\left(x\right) & \propto \prod_{i=1}^{N}G_{GTA}\left(x_{i}|x_{pa(i)}\right)\prod_{i=1}^{N}t_{i}\left(x_{i}\right)\\ \; & =\prod_{i=1}^{N}\underset{p_{GTA,i}\left(x_{i}\right)}{\underset{︸}{G_{GTA}\left(x_{i}|x_{pa(i)}\right)t_{i}\left(x_{i}\right)}}. \end{array}$$

where $p_{GTA,i}\left(x_{i}\right)≜G_{GTA}\left(x_{i}\right)t_{i}\left(x_{i}\right)$. Proceeding on such a loop-free tree graph, message passing can then be utilized to perform efficient detection during all but one iteration.

## 4. Applications into MIMO High-Order Detection

Introducing the nested variational chain for MIMO detection, it can be seen that all existing approaches employ factor substitution. As for inner approximation, MMSE and EC actually perform mean-field approximation with fully factorized distribution, while GTA performs the maximum spanning tree approximation. Finally, only EC performs factor updating, while MMSE and GTA choose to perform direct detection.

This analysis puts forward the question of whether any improvement can be achieved when one enables GTA to update its substituted factors or whether any better inner approximation can be derived for EC rather than being fully factorized. Both thoughts lead us to an idea that it is worth trying to update the GTA factors iteratively since the approximated Gaussian tree is capable of capturing correlation among symbols rather than keeping independence among them. Following this idea, an initial application of the nested variational chain can be performed. By utilizing EC as an outer approximation, an algorithm named GTA-embedded EC (GTA-EC) is proposed in the following.

### 4.1. The GTA-EC Algorithm

Given $t_{i}\left(x_{i}\right)\propto I_{x_{i}\in A}$ for i=1,…,N, the algorithm starts from the likelihood function with discrete priors as in (3), i.e.,

(25) $$\begin{array}{c} Q_{GTA-EC}\left(x\right)\propto N\left(y:\mathrm{Hx},\sigma_{n}^{2}I\right)\prod_{i=1}^{N}I_{x_{i}\in A}, \end{array}$$

which could be divided into two parts, i.e.,

(26) $$\begin{array}{cc} \; & f_{q}\left(x\right)=N\left(y:\mathrm{Hx},\sigma_{n}^{2}I\right), \end{array}$$

(27) $$\begin{array}{cc} \; & f_{r}\left(x\right)=\prod_{i=1}^{N}I_{x_{i}\in A} \end{array}$$

It is then possible to define a new distribution $q\left(x\right)$ as

(28) $$\begin{array}{c} q\left(x\right)\propto f_{q}\left(x\right)\exp\left(\gamma_{q}^{⊤}x-\frac{x^{⊤}\Lambda_{q}x}{2}\right), \end{array}$$

of which the moments can be expressed as

$$\left\{\begin{array}{cc} \; & \Sigma_{q}={\left(H^{⊤}H+\Lambda_{q}\right)}^{-1}\\ \; & \mu_{q}=\Sigma_{q}\left(\sigma_{n}^{-2}H^{⊤}y+\gamma_{q}\right) \end{array}\right.$$

Note that the pair $\left(\gamma_{q},\Sigma_{q}\right)$ acts as priors of all symbols to be updated, and that the definition of $q\left(x\right)$ actually serves as factor substitution.

To achieve moment consistency, another distribution $s\left(x\right)$ is then defined as

(29) $$s\left(x\right)\propto \exp\left(\gamma_{s}^{⊤}x-\frac{x^{⊤}\Lambda_{s}x}{2}\right),$$

where moment matching between $s\left(x\right)$ and $q\left(x\right)$ should be achieved so as to obtain $\gamma_{s}$ and $\Lambda_{s}$. The EC algorithm assumes another distribution:

(30) $$\begin{array}{cc} r\left(x\right) & \propto \exp\left(\gamma_{r}^{⊤}x-\frac{x^{⊤}\Sigma_{r}x}{2}\right)f_{r}\left(x\right), \end{array}$$

with moments derived as

$$\left\{\begin{array}{cc} \; & \gamma_{r}=\gamma_{s}-\gamma_{q}\\ \; & \Lambda_{r}=\Lambda_{s}-\Lambda_{q} \end{array}\right.$$

It can be observed that $\exp\left(\gamma_{r}^{⊤}x-\frac{x^{⊤}\Sigma_{r}x}{2}\right)$ partly in $r\left(x\right)$ actually serves as a cavity distribution of symbols by subtracting their substituted priors $\left(\gamma_{q},\Sigma_{q}\right)$.

The next step involves inner approximation. Since fully factorization for $r\left(x\right)$ neglects correlation among symbols, we instead propose utilizing the Gaussian approximation tree to perform detection according to the moments $\mu_{r}$, $\Sigma_{r}$, $\mu_{q}$, and $\Sigma_{q}$. This is because the Gaussian approximation tree may capture correlation among symbols rather than treating them independently. In this case, we define a new Gaussian tree-based distribution $g\left(x\right)$ rather than $r\left(x\right)$ as

(31) $$\begin{array}{cc} g\left(x\right) & \propto f_{r}\left(x\right)\prod_{i=1}^{N}G^{\setminus i}\left(x_{i}|x_{pa(i)}\right)=\prod_{i=1}^{N}\underset{p_{i|pa(i)}^{\prime }\left(x_{i}\right)}{\underset{︸}{G^{\setminus i}\left(x_{i}|x_{pa(i)}\right)I_{x_{i}\in A}}} \end{array}$$

where $p_{i|pa(i)}^{\prime }≜G^{\setminus i}\left(x_{i}|x_{pa(i)}\right)I_{x_{i}\in A}$ is a new distribution by attaching true priors, and the conditional distribution can be represented as

(32) $$\begin{array}{cc} \; & G^{\setminus i}\left(x_{i}|x_{pa(i)}\right)\propto exp\left\{-\frac{1}{2}\frac{{\left[\left(x_{i}-\mu_{i}^{r}\right)-\frac{\Sigma_{i,pa(i)}}{\Sigma_{pa(i),pa(i)}}\left(x_{pa(i)}-\mu_{pa(i)}\right)\right]}^{2}}{\Sigma_{i,i}^{r}-\frac{\Sigma_{i,pa(i)}}{\Sigma_{pa(i),pa(i)}}}\right\} \end{array}$$

where $\mu_{i}^{r}$ and $\Sigma_{i,i}^{r}$ for i=1,…,N are taken from $\mu_{r}$ and the diagonal of $\Sigma_{r}$, respectively, while $\mu_{i}$ and $\Sigma_{i,i}$ for i=1,…,N are taken from $\mu_{q}$ and the diagonal of $\Sigma_{q}$.

Based on $g\left(x\right)$, message passing on the Gaussian tree can then be proceeded:

(33) $$\begin{array}{c} M_{i\to pa(i)}\left(x_{pa(i)}\right)=\sum_{∼x_{i}}\;\underset{p_{i|pa(i)}\left(x_{i}\right)}{\underset{︸}{G^{\setminus i}\left(x_{i}|x_{pa(i)}\right)t_{i}\left(x_{i}\right)}}\prod_{j|pa(j)=i}\;M_{j\to i}\left(x_{i}\right) \end{array}$$

and

(34) $$\begin{array}{cc} M_{pa(i)\to i}\left(x_{i}\right) & =\sum_{∼x_{pa(i)}}\;\underset{p_{i|pa(i)}\left(x_{i}\right)}{\underset{︸}{G^{\setminus i}\left(x_{i}|x_{pa(i)}\right)t_{i}\left(x_{i}\right)}}\\ \; & \times \;\prod_{j|j\neq i,pa(j)=pa(i)}\;M_{j\to p(i)}\left(x_{pa(i)}\right)M_{pa(pa(i))\to pa(i)}\left(x_{pa(i)}\right) \end{array}$$

To achieve consistency, the distribution $s\left(x\right)$ is finally utilized once again to achieve moment matching between $g\left(x\right)$ and $s\left(x\right)$ so as to obtain $\gamma_{s}$ and $\Sigma_{s}$, and the a priori moments can be updated:

(35) $$\left\{\begin{array}{cc} \; & \Lambda_{q}^{new}=\beta \left(\Lambda_{s}-\Lambda_{r}\right)+\left(1-\beta \right)\Lambda_{q}\\ \; & \gamma_{q}^{new}=\beta \left(\gamma_{s}-\gamma_{r}\right)\;+\left(1-\beta \right)\gamma_{q} \end{array}\right.$$

The GTA-EC algorithm is concluded and depicted in detail in Algorithm 2. In step 1, the GTA-EC algorithm initiliazes the distribution $q\left(x\right)$, which behaves as an outer approximation by substituting true factors. In step 2, the inner approximation is applied to $q\left(x\right)$ by using its moments, such that a maximum spanning tree is constructed. With the derived tree structure, the algorithm repeats step 3 and step 4 over iterations such that factors can be updated by performing symbol detection and moment matching, and hard outputs can then be obtained according to the final distribution.
**Algorithm 2** The GTA-EC Algorithm**Require: **y, H, $E_{s}$ and $\sigma_{n}^{2}$. Initialize $M_{i\to pa(i)}\left(x_{pa(i)}\right)=1/\sqrt{A}$, $M_{pa(i)\to i}\left(x_{i}\right)=1/\sqrt{A}$, $\gamma_{i}=0$ and $\Lambda_{i}=E_{s}^{-1}$ for i=1,…,N.**Ensure:****(1) Step 1: Factor Substitution**.Initial $q\left(x\right)\propto f_{q}\left(x\right)\exp\left(\gamma_{q}^{⊤}x-\frac{1}{2}x^{⊤}\Lambda_{q}x\right)$.**(2) Step 2: Inner Approximation**.The maximum Gaussian spanning tree is constructed according to the initial covariance matrix such that the tree structure and relationship among symbols can be obtained.**repeat****(3) Step 3: Symbol Detection**.Obtain $r\left(x\right)$ by achieving consistency between $q\left(x\right)$ and $s\left(x\right)$, and obtain $g\left(x\right)$ according to the established tree structure and derived moments. Perform message passing in updating $M_{i\to pa(i)}\left(x_{pa(i)}\right)$ and $M_{pa(i)\to i}\left(x_{i}\right)$ according to (33) and (34), and obtain the aposteriori statistics by achieving consistency between $g\left(x\right)$ and $s\left(x\right)$.**(4) Step 4: Factor Updating**.Update $\gamma_{q}^{new}$ and $\Lambda_{q}^{new}$ such that $q^{new}\left(x\right)$ can be updated.**until** A maximum number of iterations has been achieved.**Output:** Hard outputs according to the first-order moments of the latest $q^{new}\left(x\right)$.

### 4.2. Complexity Analysis

The calculation of GTA-EC resides mainly on three parts. The first one involves the factor substitution step, which necessitates the calculation of second-order and first-order moments in (29), the same as MMSE in (16) or EC in (19). As is well known, its complexity in one iteration can be given as $O(NM^{2})$. The second part involves construction of the tree graph for inner approximation, which needs only to be initialized at the very beginning of iterations. The construction is based on Prim’s algorithm, whose complexity is $O(M^{2})$. The last part involves the calculation of message passing and factor updating. For each iteration, the major complexity lies in calculating messages in (33) and (34), each requiring the maximum likelihood detection on the conditional distribution with the cardinality of PAM constellation being $\left|A\right|=\sqrt{A}$. Since there are M−1 conditional distributions in the tree graph, the complexity can be represented as $O\left({M|A|}^{2}\right)=O\left(MA\right)$. Therefore, by defining $N_{iter}$ as the number of iterations to proceed, the total complexity can be expressed as $O\left(\left(N_{iter}+1\right)NM^{2}+M^{2}+N_{iter}MA\right)\approx O\left(\left(N_{iter}+1\right)NM^{2}\right)$ due to the reason that $NM^{2}\gg MA$ is normally satisfied in a massive MIMO system. This indicates that the complexity of GTA-EC is about $N_{iter}$ times more than that of MMSE or GTA, namely $O\left(NM^{2}\right)$. As a comparison, the complexity of EC can be expressed as $O\left(\left(N_{iter}+1\right)NM^{2}+M+N_{iter}M\sqrt{A}\right)\approx O\left(\left(N_{iter}+1\right)NM^{2}\right)$, suggesting that the complexity of GTA-EC is approximately in the same order. The less iterations one algorithm needs to perform, the less complexity it requires. In the next section, when comparing the performance of GTA-EC with EC, the number of iterations should be utilized for complexity comparison. A summary of complexity comparison is demonstrated in Table 1, in which it can be found that the total complexity is dominated by the complexity of factor substitution as well as the number of iterations.

## 5. Numerical Results

### 5.1. Simulation Parameters

In this section, the detection performance of a MIMO system is evaluated in terms of bit error rate (BER). Uncorrelated scattering flat-fading channel model is assumed with channel coefficients being modeled as complex Gaussian distributed variables that are independently generated for all antennas. During the simulation, 20,000 realizations of the channel matrix are employed with each used to send one message. As a comparison, several existing algorithm are evaluated as well such as the MMSE, GTA, and EC algorithms. And we mainly take into consideration the ’worst-case’ scenarios of load α=N/M=1 when N=M=16 and N=M=64 with high-order constellations 16-QAM, 64-QAM, and 256-QAM considered. The factor β is set as 0.2 for all algorithms, and the iteration number of EC and GTA-EC is set as 2, 4, and 6 since convergence can be achieved within six iterations.

### 5.2. Performance Evaluation

**Figure 1:**
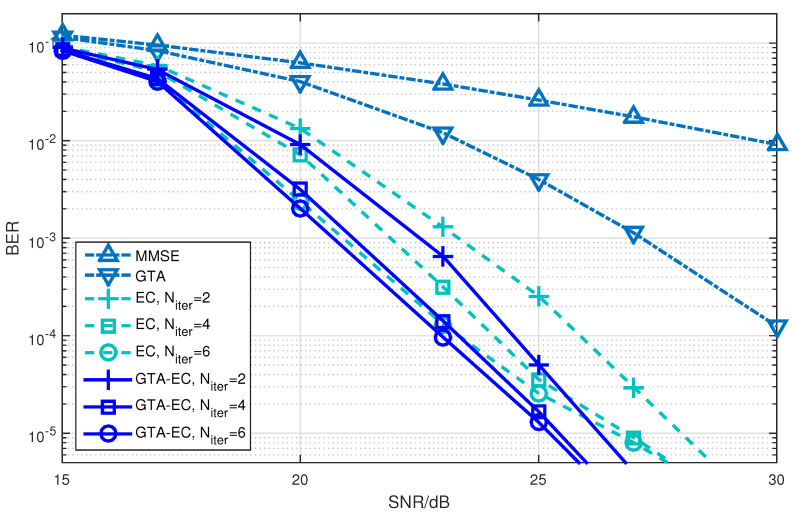
BER comparison of GTA-EC with existing algorithms when N=M=16 with 16-QAM.

**Figure 2:**
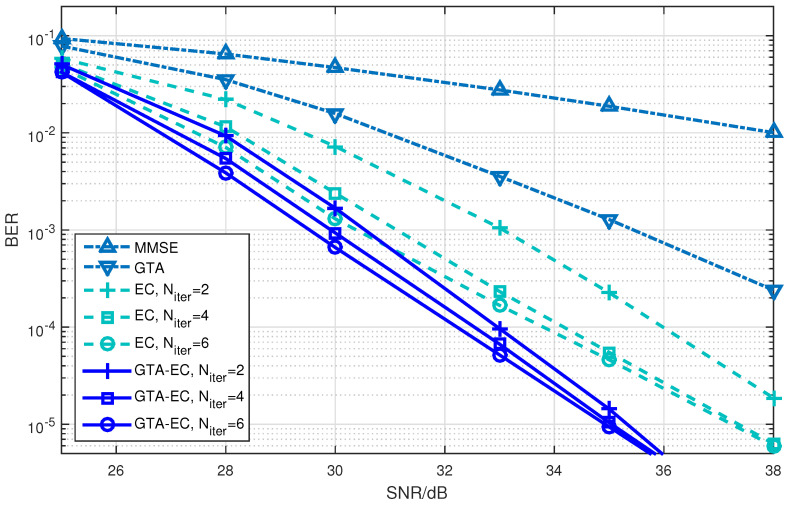
BER comparison of GTA-EC with existing algorithms when N=M=16 with 64-QAM.

**Figure 3:**
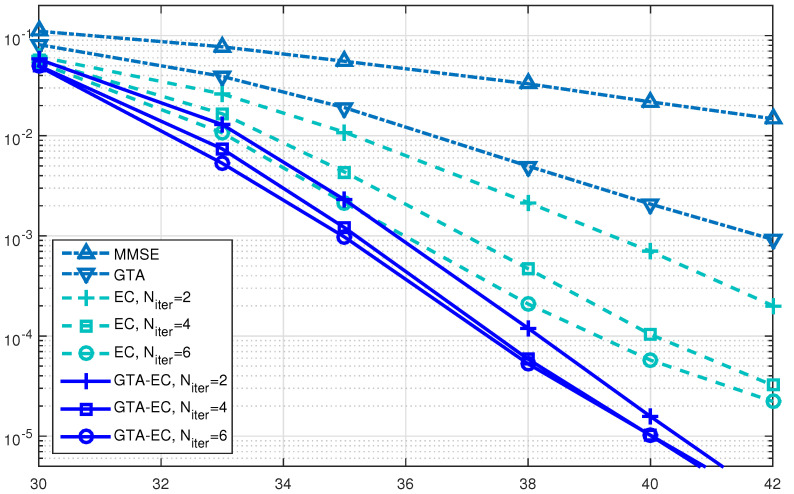
BER comparison of GTA-EC with existing algorithms when N=M=16 with 256-QAM.

Figure 1, Figure 2 and Figure 3 demonstrate the BER comparison of the GTA-EC algorithm with existing algorithms. The number of antennas deployed at both the transmitter and receiver in the system is set as N=M=16 with the constellations being 16-QAM, 64-QAM and 256-QAM, respectively. It can be found that GTA-EC outperforms EC with the same number of iterations, and that GTA-EC with four iterations outperforms EC with six iterations, indicating that GTA-EC may achieve better performances than EC does with lower complexity. While in Figure 2 and Figure 3, GTA-EC with two iterations almost exibits better performance than EC with six iterations, revealing better performance gain when high-order constellations are employed. One can further obseve that the BER slopes of GTA-EC decrease faster than that of EC, demonstrating that superior divergence gain can also be obtained by GTA-EC in a high SNR regime.

**Figure 4:**
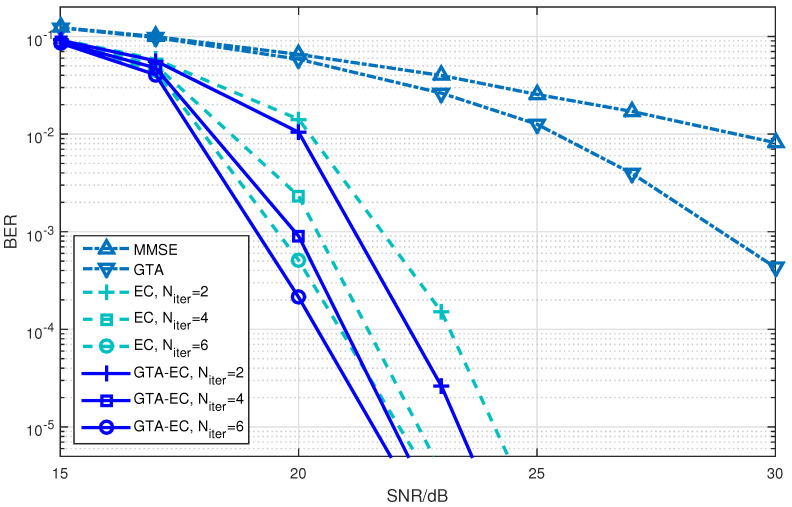
BER comparison of GTA-EC with existing algorithms when N=M=64 with 16-QAM.

**Figure 5:**
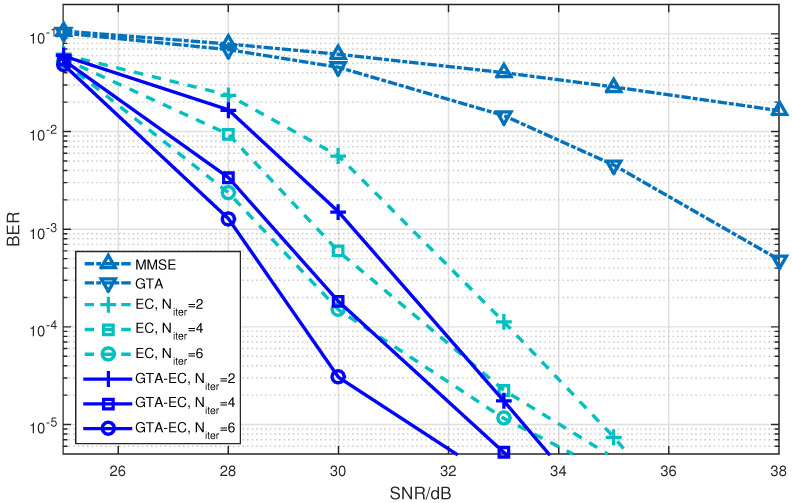
BER comparison of GTA-EC with existing algorithms when N=M=64 with 64-QAM.

**Figure 6:**
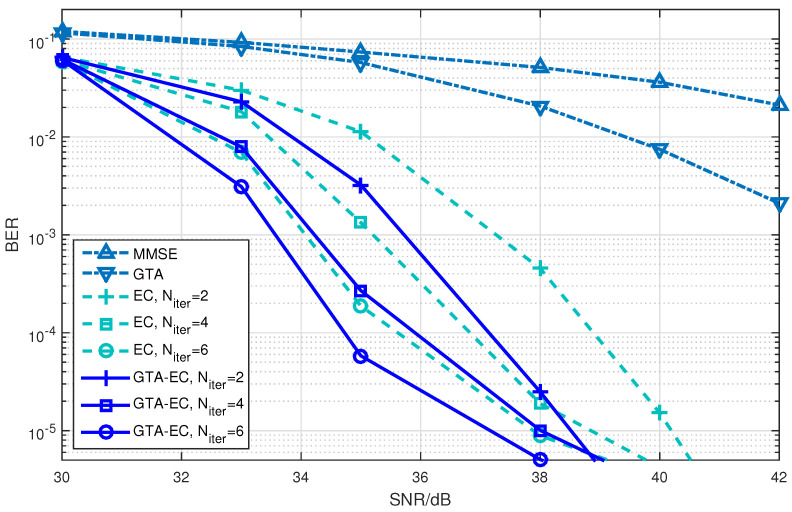
BER comparison of GTA-EC with existing algorithms when N=M=64 with 256-QAM.

Figure 4, Figure 5 and Figure 6 demonstrate a BER comparison of the GTA-EC algorithm with existing algorithms. The number of antennas deployed at both the transmitter and receiver is given as N=M=64 with the constellations being 16-QAM, 64-QAM and 256-QAM, respectively. In these figures, it can be found that GTA-EC outperforms EC with the same number of iterations, while GTA-EC with four iterations may have similar performance to that of EC with six iterations. This indicates that GTA-EC exhibits better performance than EC does at the same order of complexity or that GTA-EC presents similar performance to that of EC with lower complexity. And in Figure 4 and Figure 5, one can further observe that the BER slope of GTA-EC decreases faster than that of EC, leading to better performance in a high SNR regime.

By observing and analyzing the figures in different scenarios, we may come to conclusions about the performance comparison of GTA-EC with existing algorithms.

- On one hand, both EC and GTA-EC significantly outperform existing algorithms such as MMSE and GTA. In most scenarios, GTA-EC can obviously outperform EC with either 16-QAM, 64-QAM, or 256-QAM employed. The performance gain of GTA-EC becomes larger when high-order constellation is employed. For example, both the 64-QAM and 256-QAM cases exhibit larger gain than the 16-QAM case when employing 16 or 64 antennas. This indicates that GTA-EC has superior performance gain and is especially suitable for high-order constellations. We believe that the performance gain comes from exploiting additonal relations (correlation) among symbols rather than treating them independently.
- On the other hand, as for the complexity issue, GTA-EC with four iterations may outperform or have comparable performance to EC with six iterations, suggesting that GTA-EC requires less complexity than EC by recalling that their computational burdens are dominated by the number of iterations needed. As a result, four iterations are recommended for GTA-EC according to the simulation results, and hence the complexity of GTA-EC is approximately four times more than MMSE, indicating that it is a practical method for massive MIMO systems.

## 6. Conclusions

A nested variational chain is proposed along with an algorithm provided, which combines two asymmetic KL divergences. Introduced into MIMO systems, it can be found that several existing algorithms such as MMSE, GTA, and EC can be regarded as special cases. As initial applications for MIMO detection, an algorithm named GTA-EC is proposed with complexity analysis, and numerical results prove that it may achieve better detection performance with less complexity compared to existing algorithms. As for further research topics, it is suggested that one can find better inner approximation that may capture much more correlation among symbols by applying this framework to other detection fields, such as space code multiple access (SCMA), orthogonal time frequency space (OTFS), or low-density parity check (LDPC) decoding systems.

## Figures and Tables

**Table 1:** Comparisons of complexity.

Algorithm	Factor Substitution	Inner Approximation	Detection and Factor Updating	Total Complexity
MMSE	$O\left(NM^{2}\right)$	$O\left(M\right)$	$O\left(M\sqrt{A}\right)$	$O\left(NM^{2}\right)$
GTA	$O\left(NM^{2}\right)$	$O\left(M^{2}\right)$	$O\left(MA\right)$	$O\left(NM^{2}\right)$
EC	$O\left(\left(N_{iter}+1\right)NM^{2}\right)$	$O\left(M\right)$	$O\left(N_{iter}M\sqrt{A}\right)$	$O\left(\left(N_{iter}+1\right)NM^{2}\right)$
GTA-EC	$O\left(\left(N_{iter}+1\right)NM^{2}\right)$	$O\left(M^{2}\right)$	$O\left(N_{iter}MA\right)$	$O\left(\left(N_{iter}+1\right)NM^{2}\right)$

## Data Availability

Data are contained within the article.
